# Hepatoprotective and anti-inflammatory activities of *Plantago major* L

**DOI:** 10.4103/0253-7613.55211

**Published:** 2009-06

**Authors:** Idris Türel, Hanefi Özbek, Remzi Erten, Ahmet Cihat Öner, Nureddin Cengiz, Orhan Yilmaz

**Affiliations:** Departments of Pharmacology and Toxicology, Yüzüncu Yil University, 65300 Van-Turkey; 1 Department of Pharmacology, Yüzüncu Yil University, 65300 Van-Turkey; 2 Department of Pathology, Yüzüncu Yil University, 65300 Van-Turkey; 3 Department of Histology-Embryology, Yüzüncu Yil University, 65300 Van-Turkey

**Keywords:** Anti-inflammatory activity, hepatoprotective activity, *Plantago major* L., rat

## Abstract

**Objective:**

The aim of this study was to investigate anti-inflammatory and hepatoprotective activities of *Plantago major* L. (PM).

**Materials and Methods:**

Anti-inflammatory activity: Control and reference groups were administered isotonic saline solution (ISS) and indomethacin, respectively. Plantago major groups were injected PM in doses of 5 mg/kg (PM-I), 10 mg/kg (PM-II), 20 mg/kg (PM-III) and 25 mg/kg (PM-IV). Before and three hours after the injections, the volume of right hind-paw of rats was measured using a plethysmometer.

**Hepatoprotective Activity:**

The hepatotoxicity was induced by carbon tetrachloride (CCl4) administration. Control, CCl4 and reference groups received isotonic saline solution, CCl4 and silibinin, respectively. *Plantago major* groups received CCl4 (0.8 ml/kg) and PM in doses of 10, 20 and 25 mg/kg, respectively for seven days. Blood samples and liver were collected on the 8th day after the animals were killed.

**Results:**

*Plantago major* had an anti-inflammatory effect matching to that of control group at doses of 20 and 25 mg/kg. It was found that reduction in the inflammation was 90.01% with indomethacin, 3.10% with PM-I, 41.56% with PM-II, 45.87% with PM-III and 49.76% with PM-IV. Median effective dose (ED50) value of PM was found to be 7.507 mg/kg. Plantago major (25 mg/kg) significantly reduced the serum alanine aminotransferase (ALT) and aspartate aminotransferase (AST) levels when compared to the CCl4 group. The histopathological findings showed a significant difference between the PM (25 mg/kg) and CCl4 groups.

**Conclusion:**

The results showed that PM had a considerable anti-inflammatory and hepatoprotective activities.

## Introduction

*Plantago major* L. (PM) is a member of the Plantaginaceae family. The plant is used as folk medicine due to their hepatoprotective and anti-inflammatory properties in Turkey.[1–4]

Anti-diarrhoeal and anti-nociceptive effects of *Plantago major* were reported.[5,6] Some researchers showed that *Plantago major* L. had uterotonic action in guinea pig, prophylactic effect for mammary cancer in mice and protective effect against systemic *Streptococcus pneumoniae* infection in mice.[7–9] Ozaslan *et al.* reported that PM extract (especially 1% concentration) had inhibitive effect on Ehrlich Ascites Tumor.[10] Velasco-Lezama *et al.* showed that *Plantago major* has hematopoietic activity *in vitro*.[11] Some researchers reported that hot water extracts of *Plantago major* and *Plantago asiatica* possess a broad-spectrum of anti-leukaemia, anti-carcinoma and anti-viral activities, as well as activities which modulate cell-mediated immunity.[12] Holetz *et al*. showed that *Plantago major* and *Erythrina speciosa* presented some degree of antibacterial activity.[13] Chiang *et al*. reported that pure compounds of PM, which possess anti-viral activities are mainly derived from the phenolic compounds, especially caffeic acid.[14] Some researchers reported that PM had a rapid effect on subjective complaints and objective findings in the treatment of chronic bronchitis.[15] Chakraborty *et al*. showed that *Plantago ovata* mucilage showed better disintegrated property than the most widely used super disintegrants like sodium starch glycolate and croscarmellos sodium (Ac-di-sol) in the formulation of fast dissolving tablets.[16]

The present study had the following objectives: (1) to evaluate the anti-inflammatory activity of the methanol extract of *Plantago major* L. seeds on carrageenan-induced rat paw oedema; (2) to determine the hepatoprotective effect of the methanol extract of *Plantago major* L. seeds on CCl_4_-induced hepatotoxicity in rats.

## Materials and Methods

### Plant material

*Plantago major* L. was collected from the vicinities of Edremit-Van in September, 2007. Taxonomic identity of the plant was confirmed by Dr. Lütfü Behçet, a plant taxonomist in the Department of Biological Sciences, Yüzüncü Yil University, Van-Turkey. Voucher specimens for the plant seeds have been deposited in Pharmacology Laboratory of Yüzüncü Yil University (B-24). The dried seeds of PM were finely grounded in an electrical grinder and extracted by Soxhlet apparatus (Ildam, Turkey) with methanol (40–50°C) until completely exhausted. Methanol was evaporated under reduced pressure by a rotary evaporator (IKA-WERKE RV 05-ST rotavapor, Germany). The yield was determined as 3.02% (w/w).

### Animals

Female and male Sprague-Dawley rats weighing 150–250 g were used in these experiments. The animals were housed at room temperature (20 ± 2°C) in standard cages with standard pellet food and water *ad libitum*, and kept under controlled environment following the standard operating procedures of the animal house with the approval of animal ethics committee.

### Chemicals

Lambda-carrageenan Type IV, indomethacin and silibinin were obtained from Sigma (Steinheim, Germany), methanol and carbon tetrachloride (CCl_4_) were obtained from Merck (Darmstadt, Germany), Tween 80 was obtained from Merck (Hohenbrunn, Germany) and olive oil was obtained from Fluka (Steinheim, Germany). Lambda-carrageenan was dissolved in distilled water (w/v), silibinin and indomethacin were dissolved in ethyl alcohol (w/v), CCl_4_ dissolved in olive oil (v/v) (1: 1 dilutions) and PM was dissolved in 2% Tween 80 (w/v).

### Anti-inflammatory activity

The method of Winter *et al*. with slight modification was used.[17] Thirty-six rats of either sex were divided into six groups of six animals each. Inflammation of the hind paw was induced by injecting 0.05 ml fresh Lambda-carrageenan (phlogistic agent) into the sub-plantar surface of the right hind paw. The experimental groups were as follows (*n* = 6):

Group 1: Physiologic saline (0.9 % isotonic saline solution, ISS), 0.1 ml, po;

Group 2: Indomethacin (3 mg/kg),[18] ip;

Group 3: PM-I (5 mg/kg), ip;

Group 4: PM-II (10 mg/kg), ip;

Group 5: PM-III (20 mg/kg), ip;

Group 6: PM-IV (25 mg/kg), ip.

These doses of the extract utilized in the current study have been chosen according to Atta and Mouneir with modification.[6] The measurement of foot volume was accomplished by displacement technique using a plethysmometer (Ugo Basile 7140 plethysmometer, Italy), immediately before and three hours after the injection. The inhibition percentage of the inflammatory reaction was determined for each animal by comparison with controls and calculated by the formula:[19]

I%=[(1−(dt/dc)]×100.

where *dt* is the difference in paw volume in the drug-treated group and *dc* the difference in paw volume in the control group.

### Hepatoprotective activity

The CCl_4_ model described by Handa and Sharma and Shenoy *et al.* was used for scheduling the dose regimen.[20,21] Intraperitoneal injection of 0.8 ml/kg CCl_4_ diluted in olive oil (1: 1 dilution) was employed for inducing acute liver toxicity. The experimental groups were as follows (*n* = 6):

Group 1: Physiologic saline (0.9% isotonic saline solution, ISS), 0.1 ml, ip;

Group 2: CCl_4_: olive oil (1: 1) (0.8 ml/kg), ip;

Group 3: Silibinin 50 mg/kg and CCl_4_: olive oil (1: 1) (0.8 ml/kg), ip;

Group 4: PM (10 mg/kg) and CCl_4_: olive oil (1: 1) (0.8 ml/kg), ip;

Group 5: PM (20 mg/kg) and CCl_4_: olive oil (1: 1) (0.8 ml/kg), ip;

Group 6: PM (25 mg/kg) and CCl_4_: olive oil (1: 1) (0.8 ml/kg), ip.

The doses of the PM and silibinin utilized in the current study have been chosen according to Atta and Mouneir[6] and Horváth *et al*.,[22] respectively. All injections were applied once a day for seven days. CCl_4_, PM and silibinin were applied separately using different injectors. The animals were observed daily and any dead animals were subjected to post-mortem examination to find the cause of death. At the end of the treatment (8th day), blood samples were collected by direct cardiac puncture and the serum was used for the assay of marker enzymes, aspartate aminotransferase (AST) and alanine aminotransferase (ALT).

Body weights of the rats were measured once a day during eight days. Daily changes in body weights as percentages were recorded. The percentage of daily changes in body weights was calculated according to the following formula:

Change in body weights as

percentage = 100 × (Weight*_n_* − Weight_initial_)/Weight_initial_

Weight_initial_: measurement of first day.

Weight_n_: measurement of 2., 3., … 8 days.

The serum AST and ALT concentrations were determined with a commercial kit (Vitros) by Vitros DT60-II Autoanalyzer (USA, Rochester-New York). The livers of the experimental animals were extracted after sacrificing the animals by cervical dislocation and fixed in 10% neutral buffered-formalin prior to routine processing in paraffin-embedded blocks. Sections (4 μm thick) were cut and stained using hematoxylin-eosin (HE) stain. Histological damage was expressed using the following score system; 0: absent; +: mild; ++: moderate; +++: severe.

### Statistical analysis

All data were represented as mean ± standard error of the mean (SEM) or as percentages. Analysis of variance (ANOVA) was used for the statistical analysis of data. Tukey's HSD test (Tukey's honestly significant difference test) and LSD test (least significant difference test) were used for determining significance. Results with *P* < 0.05 were considered as statistically significant.

## Results

### Anti-inflammatory activity

Table 1 shows the results on anti-inflammatory effect of PM on carrageenan paw oedema in rats. The doses of 20 and 25 mg/kg PM caused a significant reduction in paw oedema (*P* < 0.05). As seen in Table 1, PM showed anti-inflammatory activity higher than the control group, but it did not show so strong effect as indomethacin (reference drug), which produced a significant inhibition (90.01%). It was found that reduction in the inflammation was 3.10% with PM-I, 41.56% with PM-II, 45.87% with PM-III and 49.76% with PM-IV. Median effective dose (ED_50_) value of TFG was found to be 7.507 mg/kg.

**Table 1:** Effect of *Plantago major* L. on carrageenan-induced hind paw oedema in rats (*n* = 6)

*Groups*	*Dose*	*Paw oedema (ml %)*	*Inhibition (%)*
Control (ISS)	0.1 ml	0.838 ± 0.072	-
Indomethacin	3 mg/kg	0. 084 ± 0.0^27^^a^	90.01
PM-I	5 mg/kg	0.812 ± 0.075^b^	3.10
PM-II	10 mg/kg	0.490 ± 0.074^b^	41.56
PM-III	20 mg/kg	0.453 ± 0.135^a^	45.87
PM-IV	25 mg/kg	0.421 ± 0.103^a^^c^	49.76
*F/p value*		9.140/0.000	

The values represent the mean ± S.E.M.; ED_50_: 7.507 mg/kg.; Post-hoc Tukey's HSD (honestly significant difference) test

a *P* < 0.05 with respect to control (ISS) group;

b *P* < 0.05 with respect to indomethacin group

c *P* < 0.05 with respect to PM-I group.

### Hepatoprotective activity

No difference in plasma AST and ALT levels was detected in serum from physiologic saline and silibinin injected rats (*P* > 0.05). However, significant increases in serum ALT and AST levels were observed in rats administered with CCl_4_ (*P* < 0.05). The dose of PM (25 mg/kg) treated group had significantly lower levels of AST and ALT when compared with the CCl_4_ group (*P* < 0.05). The other PM groups had no difference in serum ALT and AST levels as compared to the CCl_4_ animals, as shown in Table 2.

**Table 2:** Effect of *Plantago major* L. on serum AST and ALT levels in rats (n = 6)

*Groups*	*ALT Serum (U/L)*	*AST Serum (U/L)*
Control (ISS)	43.50 ± 3.03	157.00 ± 12.39
CCl_4_	1166.86 ± 86.81^a^	2030.86 ± 196.99
Silibinin	150.67 ± 34.19^b^	549.33 ± 71.35^b^
PM (10 mg/kg)	1521.20 ± 244.15^a^^b^^c^	1987.20 ± 386.33^a^^c^
PM (20 mg/kg)	1411.20 ± 445.86^a^^b^^c^	1347.60 ± 415.24^a^^c^
PM (25 mg/kg)	663.00 ± 175.69^a^^c^^d^^e^	610.00 ± 124.82^b^^d^^e^
F/p Value	13.860/0.000	16.776/0.000

The values represent the mean ± S.E.M.; Post-hoc LSD (least significant difference) test

a *P* < 0.05 with respect to control (ISS) group;

b *P* < 0.05 with respect to CCl_4_ group;

c *P* < 0.05 with respect to silibinin group.

d *P* < 0.05 with respect to PM (10 mg/kg) group.

e *P* < 0.05 with respect to PM (20 mg/kg) group.

### Histopathological examination

In control group (ISS) and silibinin group, liver sections showed normal hepatic parenchyma and stroma. Liver hepatocyte cords, sinusoids and stroma were histologically normal.

**Figure 1:**
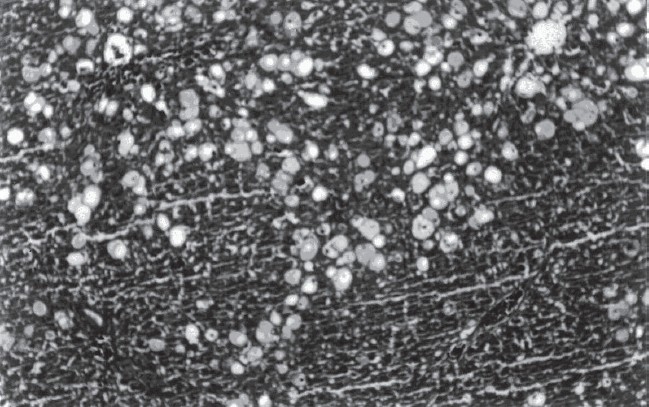
CCl4-induced hepatotoxicity in rats (numerous ballooned hepatocytes are seen in the liver) (H&E, ×100)

**Figure 2:**
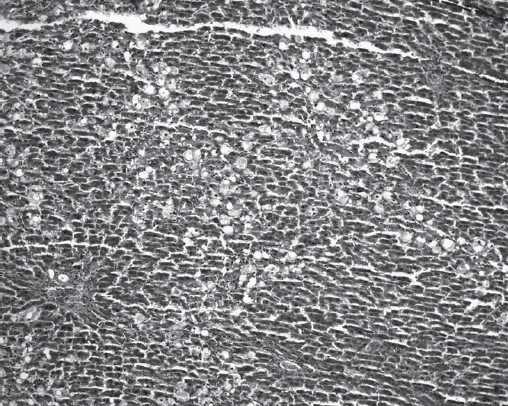
Numerous ballooned hepatocytes are seen in the liver of the PM (10 mg/kg) group (H&E, ×100)

**Figure 3:**
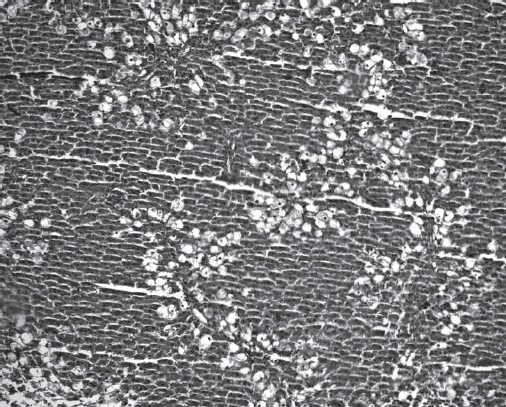
Numerous ballooned hepatocytes are seen in the liver of the PM (20 mg/kg) group (H&E, ×100)

**Figure 4:**
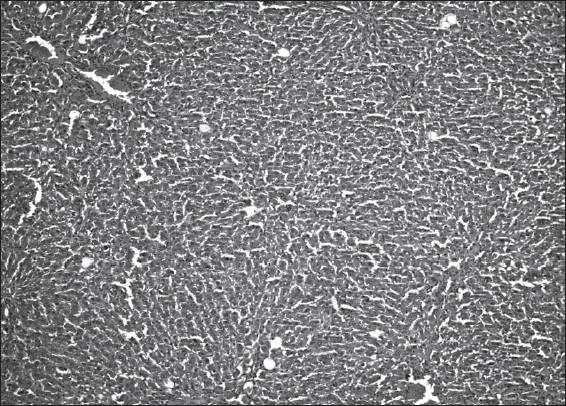
A few ballooned hepatocytes are seen in the liver of the PM (25 mg/kg) group (H&E, ×100)

Histopathological examination demonstrated that CCl_4_ (compared to ISS control group) induced ballooning degeneration, centrilobular necrosis, bridging necrosis and apoptosis (acidophilic change) in hepatocytes [Table 3]. Ballooned hepatocytes were of different sizes and much larger than normal hepatocytes and occasionally appeared as confluent areas [Figure 1]. *Plantago major* L. (10 and 20 mg/kg) treated livers did not show any significant recovery [Figures 2 and 3]. *Plantago major* L. (25 mg/kg) or silibinin-treated livers showed significant recovery. These changes were minimized by PM (25 mg/kg) or silibinin treatment [Figure 4].

**Table 3:** Effect of *Plantago major* L. on histopathological changes in the liver of the study groups*

*Groups*	*Microscopic observation*		
	
	*Ballooning degeneration and steatosis*	*Apoptosis and/or necrosis of hepatocytes*	*Bridging necrosis*
ISS	0	0	0
CCl_4_	+++	+++	+++
Silibinin	+	+	+
PM 10 mg/kg	+++	+++	+++
PM 20 mg/kg	+++	+++	+++
PM 25 mg/kg	++	+	+

0: absent

+ : mild

++ : moderate

+++ : severe.

The effects of PM on the body weight of CCl_4_-intoxicated rats were as follows: Group 1 (ISS) 6.38%, group 2 (CCl_4_) −14.35%, group 3 (silibinin) −10.27%, group 4 (PM 10 mg/kg) −12.16%, group 5 (PM 20 mg/kg) −10.10%, group 6 (PM 25 mg/kg) −8.28%. The daily body weight changes as percentages indicated that CCl_4_ group had a significant reduction in weight compared to the control group. This reduction was also observed in PM and silibinin groups [Table 4].

**Table 4:** The effects of PM on the body weight of CCl_4_-intoxicated rats

*Groups*	*Change in body weights (%)*
Control (ISS)	6.38
CCl_4_	−14.35
Silibinin	−10.27
PM (10 mg/kg)	−12.16
PM (20 mg/kg)	−10.10
PM (25 mg/kg)	−8.28

## Discussion

In this study, we used CCl_4_-induced liver toxicity that is frequently used as a model to study hepatoprotective activity of drugs.[23] The effectiveness of PM in inflammation and hepatotoxicity has been especially emphasized among some traditional claims.[1–4] Therefore, we studied PM pharmacologically and toxicologically for their above-mentioned properties.

The inhibition percentage of the inflammatory reaction and biochemical and histopathological results showed that methanol extract of PM had anti-inflammatory and hepatoprotective activities. The anti-inflammatory and hepatoprotective effects of the PM extract may be due to their content listed above. Some researchers reported that PM contains biologically active compounds such as alkaloids, polysaccharides, lipids, caffeic acid derivatives, flavonoids, iridoid glycosides, terpenoids, fatty acids and some of their structural derivatives.[24,25] Ringbom *et al*. showed that several of the natural fatty acids and triterpenoids as well as all of the semi-synthetic thioether-containing fatty acids inhibited COX-2-catalyzed prostaglandin biosynthesis, where alpha-LNA and compound 2 showed selectivity toward COX-2.[25,26] As known COX-2 is the major source of prostanoids formed in inflammation.[27] The anti-inflammatory effect of the PM may be due to their COX-2 inhibitory effect.

The hepatotoxic effects of CCl_4_ are due to its enzymatic activation to trichloromethyl (CCl3^·^) free radical, which is turn disrupts the structure and function of lipid and protein macromolecules in the membranes of the cell organelles, and induces microsomal lipid peroxidation leading to fatty liver.[28–31] In this respect, hepatocyte damages following acute CCl_4_ exposure is abrogated in experimental animals pretreated with antioxidants, such as vitamin E, demonstrating the role of oxidative activity of the trichloromethyl radical metabolite.[32] Bol'shakova *et al.* reported that PM extract had anti-oxidant effect.[31] The hepatoprotective effect of the PM extract may be due to their anti-oxidant effect.

It is concluded that the methanol extract of PM seeds have anti-inflammatory and hepatoprotective effects. The present results of the study support the traditional use of PM in inflammation and hepatotoxicity. Further studies are needed to better evaluate these activities and the anti-inflammatory and hepatoprotective potential of PM.
